# Cholecystohepaticogastrostomy: novel endoscopic gallbladder drainage technique to prevent acute cholecystitis following endoscopic ultrasound-guided biliary drainage

**DOI:** 10.1055/a-2462-1962

**Published:** 2024-11-26

**Authors:** Juan Alfonso Maclang Mendoza, Yu-Ting Kuo, Chen-Ling Peng, Hsiu-Po Wang

**Affiliations:** 1 38006 Division of Endoscopy, Department of Integrated Diagnostics and Therapeutics, National Taiwan University Hospital, Taipei, Taiwan; 2 604409 Surgery, Bicol Medical Center, Naga City, Philippines; 3 504608 Section of Surgical Endoscopy and Minimally Invasive Surgery, Department of Surgery, Rizal Medical Center, Pasig, Philippines; 4 38005 Internal Medicine, National Taiwan University College of Medicine, Taipei, Taiwan; 5 38006 Division of Gastroenterology and Hepatology, Department of Internal Medicine, National Taiwan University Hospital, Taipei, Taiwan

For malignant distal biliary obstruction (DBO), placing a stent in an antegrade manner across the obstruction and papilla, followed by endoscopic ultrasound-guided hepaticogastrostomy (EUS-HGS), establishes dual biliary drainage pathways and may prolong stent patency 1 . However, acute cholecystitis can occur after biliary drainage with a fully covered self-expandable metal stent (FCSEMS) in cases of DBO 2 . We present a novel endoscopic approach for gallbladder drainage via the HGS tract, offering a viable option for high-risk patients who develop acute cholecystitis following FCSEMS placement.

A 72-year-old woman with advanced pancreatic adenocarcinoma presented with fever, chills, and hyperbilirubinemia. She had previously undergone EUS-guided gastrojejunostomy for gastric outlet obstruction and EUS-HGS for DBO 1 month prior. Imaging revealed dilated intrahepatic ducts and common bile duct, as well as gallbladder distention, suggesting biliary infection due to HGS stent occlusion. Upon admission, EUS-HGS was performed, and biliary access was obtained via the HGS route, with aspiration of purulent bile. Following successful cystic duct cannulation, selective gallbladder cannulation was confirmed through contrast injection ( Fig. 1 , Video 1 ).

**Fig. 1:**
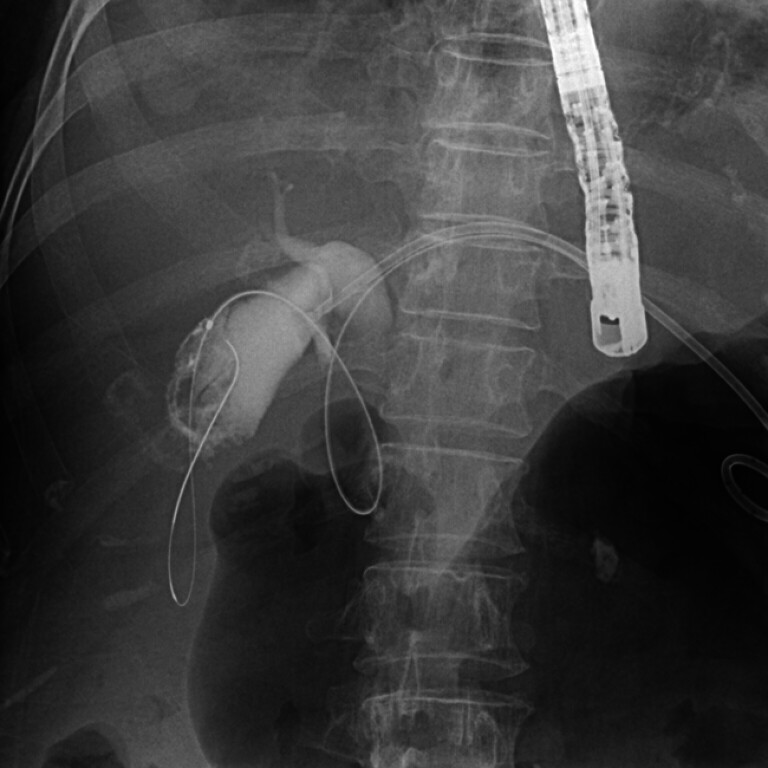
Biliary access was achieved through the hepaticogastrostomy route, and successful cannulation of the cystic duct and gallbladder was confirmed by contrast injection.

Cholecystohepaticogastrostomy was performed via the hepaticogastrostomy route following endoscopic ultrasound-guided hepaticogastrostomy with antegrade stenting to prevent cholecystitis.Video 1

A plastic stent (7-Fr diameter; 18 cm length; Through & Pass double-pigtail stent; Gadelius Medical, Tokyo, Japan) was placed between the gallbladder and stomach ( Fig. 2 ). Additionally, an FCSEMS (10 mm diameter; 7 cm length; SciTech Inc., Seoul, Korea) was placed across the biliary obstruction and the major duodenal papilla following balloon dilation of the HGS tract ( Fig. 3 ). Subsequently, an HGS stent (7-Fr diameter; 14 cm length; Through & Pass Type IT; Gadelius Medical) was placed between the intrahepatic duct and the stomach ( Fig. 4 ). The patient’s symptoms improved, and she was discharged in stable condition.

**Fig. 2:**
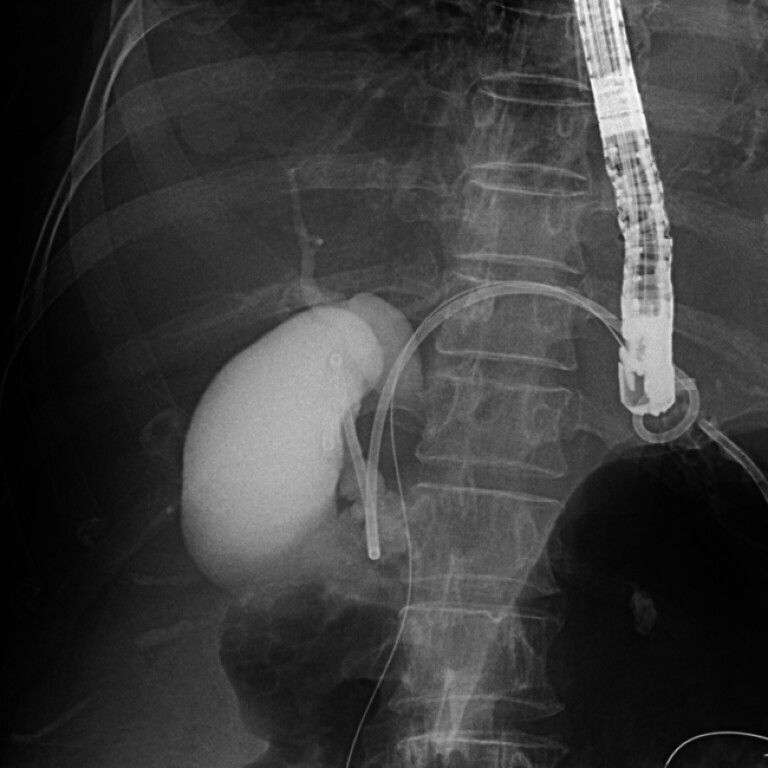
A plastic stent (7-Fr in diameter; 18 cm in length; Through & Pass double-pigtail stent; Gadelius Medical, Tokyo, Japan) was placed between the gallbladder and the stomach.

**Fig. 3:**
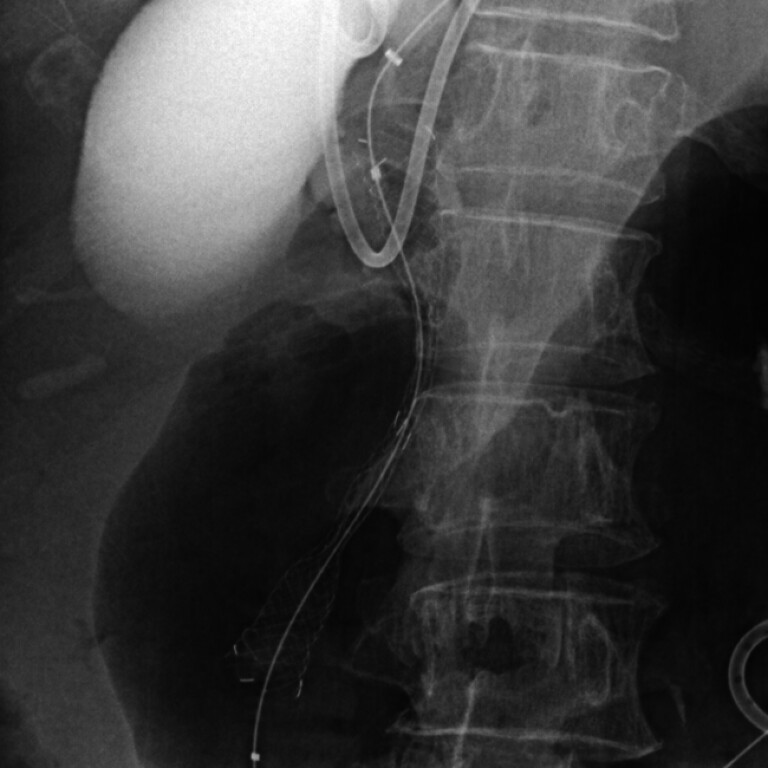
A fully covered metal stent (10 mm in diameter; 7 cm in length; SciTech Inc., Seoul, Korea) was placed antegradely across the biliary obstruction and major duodenal papilla.

**Fig. 4:**
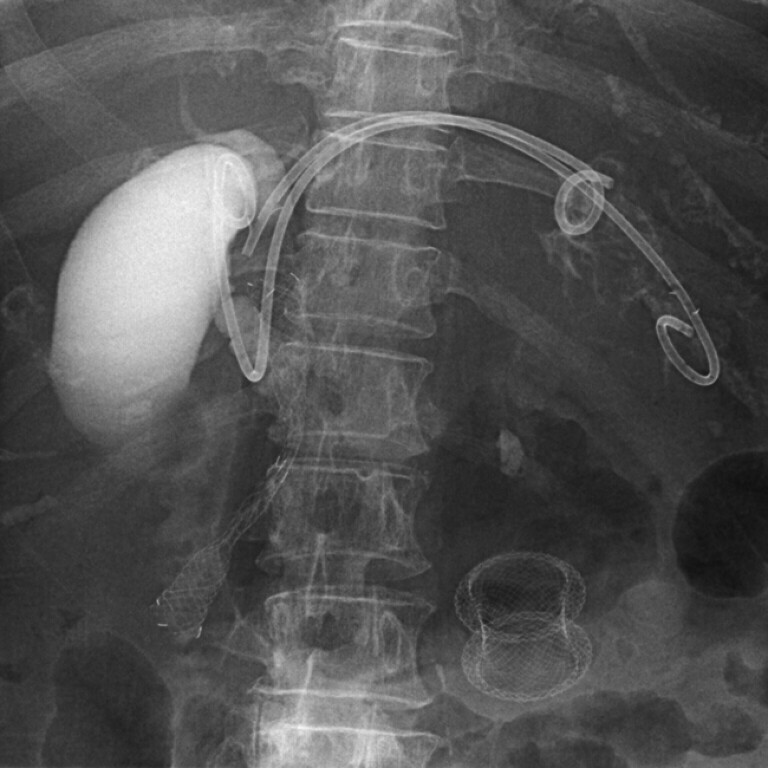
The hepaticogastrostomy stent (7-Fr in diameter; 14 cm in length; Through & Pass Type IT; Gadelius Medical, Tokyo, Japan) for additional drainage was placed between the intrahepatic duct and the stomach.

This novel gallbladder drainage technique via the HGS tract broadens treatment options for high-risk patients at risk of acute cholecystitis following EUS-HGS with FCSEMS.

Endoscopy_UCTN_Code_TTT_1AR_2AZ

## Notes

Endoscopy E-Videos https://eref.thieme.de/e-videos
*E-Videos* is an open access online section of the journal *Endoscopy* , reporting on interesting cases and new techniques in gastroenterological endoscopy. All papers include a high-quality video and are published with a Creative Commons CC-BY license. Endoscopy E-Videos qualify for HINARI discounts and waivers and eligibility is automatically checked during the submission process. We grant 100% waivers to articles whose corresponding authors are based in Group A countries and 50% waivers to those who are based in Group B countries as classified by Research4Life (see: https://www.research4life.org/access/eligibility/ ). This section has its own submission website at https://mc.manuscriptcentral.com/e-videos .
